# Leveraging multi‐modal data for individualised risk prediction for dementia

**DOI:** 10.1002/alz70856_102784

**Published:** 2025-12-25

**Authors:** Sara Calhas, Yue Liu, Sheena Waters, Oleg Blyuss, Charles R Marshall, Petroula Proitsi

**Affiliations:** ^1^ Centre for Preventive Neurology, Wolfson Institute of Population Health, Queen Mary University of London, United Kingdom, London, London, United Kingdom; ^2^ Queen Mary University of London, London, Greater London, United Kingdom; ^3^ Centre for Cancer Screening, Prevention and Early Diagnosis, Queen Mary University of London, London, London, United Kingdom; ^4^ Centre for Preventive Neurology, Wolfson Institute of Population Health, Queen Mary University of London, London, United Kingdom

## Abstract

**Background:**

Dementia is a serious global health challenge, affecting over 50 million people worldwide. Modifiable risk factors and emerging disease‐modifying treatments underscore the importance of early diagnosis. Dementia diagnosis is complex and typically occurs when symptoms emerge, which may be too late as brain damage may have occurred. The increasing availability of multimodal data and powerful machine learning algorithms opens new avenues for detecting individualised dementia risk in the preclinical stage. This research aims to identify cost‐effective, non‐invasive dementia risk signatures, such as blood‐based biomarkers for dementia risk prediction.

**Method:**

This project used data from the UK Biobank. There are two stages to the project. The first stage of the project included participants with baseline proteomic and metabolomic data, comprising 872 dementia cases and 29,006 controls, with a follow‐up period of 15.2‐years. A total of 3,241 molecules were explored. The primary focus was on machine learning to predict all‐cause dementia. Several classifiers were tested to predict all‐cause dementia at three thresholds: overall dementia prediction, dementia occurring 5 to 10 years from baseline, and more than 10 years from baseline. The second phase of the project will investigate amongst the same cohort whether the top predictor risk signatures differ by dementia sub‐type (e.g., all‐cause dementia, Alzheimer's disease and vascular dementia) and how do risk profiles differ across ethnic groups and gender.

**Results:**

XGBoost emerged as the top classifier in the first part of the project, demonstrating strong predictive power with ROC AUC scores of 0.85 or above for overall dementia prediction, as well as for predicting dementia 5 to 10 years and more than 10 years from baseline. The project offered new insights into promising plasma biomarkers for the preclinical and early diagnosis of dementia. Previously identified blood‐based biomarkers were validated, and new biomarkers were discovered.

**Conclusion:**

This work provides novel insights into specific proteins crucial for assessing individualised risk prediction for dementia and early diagnosis. The study confirmed machine learning as a viable method to help realise the benefits of preclinical dementia risk prediction. It will also shed light into differences per dementia sub‐type and how risk profiles differ across ethnic groups and gender.

